# Moral Distress, Mental Health, and Risk and Resilience Factors Among Military Personnel Deployed to Long-Term Care Facilities During the COVID-19 Pandemic: Research Protocol and Participation Metrics

**DOI:** 10.2196/44299

**Published:** 2023-11-06

**Authors:** Anthony Nazarov, Deniz Fikretoglu, Aihua Liu, Jennifer Born, Kathy Michaud, Tonya Hendriks, Stéphanie AH Bélanger, Minh T Do, Quan Lam, Brenda Brooks, Kristen King, Kerry Sudom, Rakesh Jetly, Bryan Garber, Megan Thompson

**Affiliations:** 1 MacDonald Franklin Operational Stress Injury Research Centre Lawson Health Research Institute London, ON Canada; 2 Department of Psychiatry Western University London, ON Canada; 3 Department of Psychiatry and Behavioural Neurosciences McMaster University Hamilton, ON Canada; 4 HumanSystems Inc Guelph, ON Canada; 5 Defence Research and Development Canada Department of National Defence Toronto, ON Canada; 6 Director General Military Personnel Research and Analysis Department of National Defence Ottawa, ON Canada; 7 Royal Military College of Canada Kingston, ON Canada; 8 Directorate of Mental Health Canadian Forces Health Services Department of National Defence Ottawa, ON Canada; 9 Department of Health Sciences Carleton University Ottawa, ON Canada; 10 Dalla Lana School of Public Health University of Toronto Toronto, ON Canada

**Keywords:** mental health, military, Canadian Armed Forces, operational organization, logistics support, health care, moral distress, moral injury, deployment, risk factors, COVID-19, quarantine, readiness, well-being, resilience, long-term care facility, centre de soins de longue durée, survey, older adult, qualitative interviews, quantitative

## Abstract

**Background:**

The earliest days of the COVID-19 pandemic in Canada were marked by a significant surge in COVID-19 cases and COVID-19–related deaths among residents of long-term care facilities (LTCFs). As part of Canada’s response to the COVID-19 pandemic, Canadian Armed Forces (CAF) personnel were mobilized for an initial emergency domestic deployment to the hardest-hit LTCFs (Operation LASER LTCF) to support the remaining civilian staff in ensuring the continued delivery of care to residents. Akin to what was observed following past CAF international humanitarian missions, there was an expected increased risk of exposure to multiple stressors that may be psychologically traumatic and potentially morally injurious in nature (ie, related to core values, eg, witnessing human suffering). Emerging data from health care workers exposed to the unprecedented medical challenges and dilemmas of the early pandemic stages also indicated that such experiences were associated with increased risk of adverse mental health outcomes.

**Objective:**

This study aims to identify and quantify the individual-, group-, and organizational-level risk and resilience factors associated with moral distress, moral injury, and traditional mental health and well-being outcomes of Operation LASER LTCF CAF personnel. This paper aimed to document the methodology, implementation procedures, and participation metrics.

**Methods:**

A multimethod research initiative was conducted consisting of 2 primary data collection studies (a quantitative survey and qualitative interviews). The quantitative arm was a complete enumeration survey with web-based, self-report questionnaires administered at 3 time points (3, 6, and 12 mo after deployment). The qualitative arm consisted of individual, web-based interviews with a focus on understanding the nuanced lived experiences of individuals participating in the Operation LASER LTCF deployment.

**Results:**

CAF personnel deployed to Operation LASER LTCF (N=2595) were invited to participate in the study. Data collection is now complete. Overall, of the 2595 deployed personnel, 1088 (41.93%), 582 (22.43%), and 497 (19.15%) responded to the survey at time point 1 (3 mo), time point 2 (6 mo), and time point 3 (12 mo) after deployment, respectively. The target sample size for the qualitative interviews was set at approximately 50 considering resourcing and data saturation. Interest in participating in qualitative interviews surpassed expectations, with >200 individuals expressing interest; this allowed for purposive sampling across key characteristics, including gender, rank, Operation LASER LTCF role, and province. In total, 53 interviews were conducted.

**Conclusions:**

The data generated through this research have the potential to inform and promote better understanding of the well-being and mental health of Operation LASER LTCF personnel over time; identify general and Operation LASER LTCF–specific risk and protective factors; provide necessary support to the military personnel who served in this mission; and inform preparation and interventions for future missions, especially those more domestic and humanitarian in nature.

**International Registered Report Identifier (IRRID):**

DERR1-10.2196/44299

## Introduction

### Background

As part of Canada’s response to the COVID-19 pandemic, Canadian Armed Forces (CAF) personnel were deployed on Operation LASER to support various aspects of the civilian health care sector in addressing and mitigating the impact of the COVID-19 pandemic across Canada. The scope of the mission expanded as the pandemic progressed, with activities that included contact tracing, CAF personnel vaccinations, COVID-19 testing at land ports of entry, and the subsequent deployment of intensive care unit nurses in several Canadian provinces.

The earliest days of the pandemic were marked by a significant surge in COVID-19 cases and COVID-19–related deaths among long-term care facility (LTCF) residents. In Canada, 3% of COVID-19 cases and 43% of COVID-19–related deaths occurred in LTCF residents, primarily in the provinces of Ontario and Québec [1]. The civilian staff were also falling ill with COVID-19 in high numbers and were unable to adequately mitigate the unprecedented challenges brought upon by the pandemic. Hence, on April 15, 2020, the CAF received a request for assistance to support the provision of care to residents of select LTCFs (also known as *centres d’hébergement et de soins de longue durée*) in the provinces of Québec and Ontario [2].

As part of this initial Operation LASER response, which occurred between April 2020 and July 2020, involved CAF personnel (ie, medical and support personnel, as well as command teams) were deployed to support the hardest-hit LTCFs: 47 *centres d’hébergement et de soins de longue durée* in Québec and 7 LTCFs in Ontario (all located in the Greater Toronto Area). The goal of this initial emergency deployment, hereafter referred to as *Operation LASER LTCF*, was to support the existing civilian staff in ensuring the safety of residents, maintain adequate staffing, and assist with infection control. The dire circumstances experienced in certain LTCFs were reported by the media and in CAF reports [3,4]. Similarly, anecdotal information was provided to our research team by CAF leadership.

These various reports highlighted that personnel deployed in support of the LTCFs may be exposed to multiple stressors that may be psychologically traumatic and potentially morally injurious (ie, related to important values) in nature (eg, witnessing human suffering); this is akin to what was observed in past CAF international humanitarian missions and recent experiences of health care workers (ie, medical challenges and dilemmas) during the early stages of the pandemic. More uniquely to Operation LASER, especially in the earliest days of the crisis, the COVID-19 pandemic posed a serious health and economic threat to people in Canada, not to people in a far-off land. As such, it held greater implications for the loved ones of Operation LASER personnel than perhaps any other domestic mission to date. Moreover, understanding concerning the lethality and transmissibility of the virus was unfolding and being updated on an almost daily basis at that time. Finally, the deployment was focused on assisting the most sick and vulnerable Canadian older adult patients and residents of LTCFs, a population that even seasoned CAF medical personnel were largely unfamiliar with. This combination of traditional and new stressors meant that the initial Operation LASER deployment was unique in many ways. Drawing on previous literature, such psychologically traumatic and morally injurious stressors may increase individuals’ risk of adverse mental health outcomes such as posttraumatic stress disorder (PTSD), depression, and moral injury [5-9].

Previous deployments where CAF personnel were exposed to such psychological stressors were typically international, which came with inherent logistical challenges related to implementing timely, comprehensive, and secure research data collection [10]. These are typically overcome by a rigorous chain of command that allows for direct and efficient communication between researchers and the military chain of command. Although Operation LASER was a domestic deployment, the same challenges were encountered and mitigated by the CAF Surgeon General, Canadian Joint Operations Command (CJOC) chain of command, and the Department of National Defence (DND). Understanding the challenges facing personnel in this deployment, the Surgeon General and Chief of Military Personnel, with the approval and facilitation of CJOC, requested research investigating the individual-, group-, and organizational-level risks, resilience factors, and mental health outcomes of Operation LASER LTCF personnel. In response, a multiorganizational collaborative Operation LASER research team was established to comprehensively capture and better understand the impact of this deployment on the well-being of Operation LASER LTCF personnel.

### The Structure of the Initial Operation LASER LTCF Deployment

Operation LASER LTCF personnel were typically divided into teams comprising 1 senior medical authority and a number of medical workers and support personnel. They answered to various command teams from CJOC and Canadian Forces Health Services Group (CF H Svcs Gp), as well as Land Force Command. The total number of serving military personnel ranged from 14 to 60 per facility, and they liaised with and supported the civilian medical and administrative staff on-site. In accordance with provincial health care regulations, their responsibilities included accompanying and assisting residents with daily tasks (eg, building and personal hygiene and feeding), preparing and distributing medical products, preparing meals, and maintaining medical and nonmedical equipment. To limit the potential spread of COVID-19 to their social networks, most Operation LASER LTCF personnel stayed in hotels for the duration of their deployment. Through initial discussions with CJOC, CF H Svcs Gp, and Land Force Command, the research team identified 4 overarching duty types: group 1 (Operation LASER 1) comprised medical and health care personnel who worked inside the LTCFs, group 2 (Operation LASER 2) comprised nonclinical personnel who worked inside the LTCFs (ie, provided support to clinical personnel inside the LTCFs), group 3 (Operation LASER 3) comprised nonclinical personnel who did not enter LTCFs (eg, outside general duties), and group 4 (Operation LASER 4) comprised personnel who provided support roles involved in Operation LASER (eg, Headquarters or Command, support, resupply, Company Quarters, Military Police, and clinical leadership not embedded inside the LTCFs). Before deploying to the LTCFs, Operation LASER LTCF personnel received medical briefings and were trained to use appropriate personal protective equipment (PPE) according to the level of protection required for the tasks performed or depending on the availability of the material, keeping in mind the quickness of the CAF response to the pandemic. They also received training on the physical needs and psychological and spiritual reactions that might be experienced by personnel in the context of their Operation LASER LTCF work and ways to maintain resilience and well-being.

Most of the Operation LASER LTCF deployment occurred between April 20, 2020, and June 26, 2020, for Québec and between April 27, 2020, and July 3, 2020, for Ontario [2], although many military personnel saw their engagement continue until August 31, 2020. However, germane to the study and this paper, at the time of the study protocol development (between April 2020 and May 2020), the situation was still unfolding, and certain aspects of Operation LASER LTCF were still unknown or in flux. This presented the research team with particular challenges in identifying the optimal study design for this initiative, all under tight time frames (ie, because of incomplete awareness of Operation LASER LTCF details and changes to Operation LASER LTCF parameters that were assumed to be static; for more information on specific challenges and lessons learned, refer to the study by Fikretoglu et al [11]). For instance, the deployment start date and end date were not identical for all personnel; start and end dates depended on LTCF location, and staffing requirements may have fluctuated across time (requiring reallocation of personnel to more than one Operation LASER role). Following deployment, Operation LASER LTCF personnel should have participated in a 3-day decompression session, which included additional resilience training; they then should have undergone a 14-day isolation period. However, reports from CJOC and CF H Svcs Gp indicated that the redeployment processes were not consistent across Operation LASER LTCF personnel in different roles and LTCFs. These nuances required careful data collection, protocol adjustments from the first to the third iteration of the web-based survey, and analytical considerations throughout to allow for the most accurate characterization of the sample.

### Risk and Resilience Factors and Mental Health Outcomes in Military Personnel

There is extensive research enumerating the factors that may affect short- and long-term psychological well-being and mental health outcomes following exposure to psychological trauma [12,13]. We approached the research questions by applying and testing key constructs from previous literature to the Operation LASER LTCF context and integrating them with the emerging literature on moral distress and injury. For instance, certain personal background characteristics, health status variables, and occupational roles may place military personnel at greater risk of experiencing negative reactions following Operation LASER LTCF [7,14,15]—those with prior trauma or preexisting physical or mental health conditions, those who become infected and enter extended self-isolation and quarantine, those with lower income, visible minority groups, and women may be at greater risk of adverse mental health outcomes [13]. In addition, individual-level factors such as exposure to stressful deployment experiences and maladaptive coping styles may also be associated with poorer outcomes [7,16].

Regarding stressful deployment experiences, we expected that, although some of these would be organizationally related and be commonly experienced in any deployment regardless of its nature (eg, logistical issues), other Operation LASER LTCF–specific experiences would be linked to fundamental values that an individual holds dear (eg, the need to provide care to the sick and vulnerable). Thus, we anticipated that at least some Operation LASER LTCF personnel would encounter morally laden experiences that could engender significant amounts of what is termed moral distress. Such moral distress can lead to moral injury (ie, profound and lingering psychological distress that is particularly associated with intense value conflicts [17]) and has been demonstrated to be a risk factor for depression and PTSD in CAF personnel [7,18]. Recent research with health care workers exposed to morally distressing experiences during the COVID-19 pandemic highlights the strong association between moral distress and adverse mental health outcomes, including depressive symptoms and burnout [19]. The increased recognition of moral injury as a clinically important construct [20] and the challenges it poses during help seeking and treatment [21] further underscore the necessity to understand this issue and identify opportunities for early intervention, prevention, and support.

Other research suggests that many psychosocial dimensions at the social, unit, leader, and organizational levels may also influence the effect of stressful experiences on the well-being of military personnel. Specifically, findings sourced from previous DND Human Dimensions of Operations surveys have demonstrated that poor social support, as well as less trust in units and leaders and lower feelings of relatedness to others on the mission, are more likely to be associated with poorer psychological well-being and mental health outcomes (Michaud K, unpublished data, December 2021). Past research with CAF personnel has documented gaps in mental health service use [22,23] and several unique barriers to help seeking among CAF members [24]. Moreover, the literature also demonstrates that military personnel with prior experience, training, and preparation (both for crisis work and psychological reactions) and adequate social support from personal and organizational sources (during and after deployment) fare better after stressful experiences [12,25]. Finally, those who are able to derive a sense of meaning and accomplishment as a result of the mission may be better protected from negative reactions and more likely to experience positive outcomes such as posttraumatic growth [12,25].

Given the many stressors that appear inherent to the Operation LASER LTCF deployment, lessons concerning risk and protective factors and effective preparation to promote psychological well-being following crisis work needed to be captured.

### Research Objectives

The research objectives were as follows:

- Identify and quantify the individual-, group-, and organizational-level risk and resilience factors associated with moral distress, moral injury, and traditional mental health and well-being outcomes of Operation LASER LTCF CAF personnel and, to the extent possible, determine any changes that may occur over time.
- Gain a lived experience perspective through in-depth discussions with Operation LASER LTCF participants to develop a deeper understanding of their deployment experiences. Primary areas of exploration included views regarding particular risk (eg, Operation LASER LTCF COVID-19–specific stressors) and resilience (eg, predeployment Operation LASER LTCF training, prior CAF resilience training, and adaptive coping strategies) factors and the effects of these on mental health, well-being, and recovery.
- Identify lessons that can be used to inform the decisions of senior commanders in the event of similar future deployments.

In this paper, we document the protocol for this multimethod initiative (ie, composed of quantitative surveys and qualitative interviews) [26]. Specifically, we report on the study design, sampling details, measures used, sampling weight creation, and study implementation procedures. Considering that the data collection is complete, we also report on participation profiles and metrics, which provide insights into the nature of the sample and may be useful in guiding the protocol development of future research in this field and population.

## Methods

### Study Design

**Figure 1:**
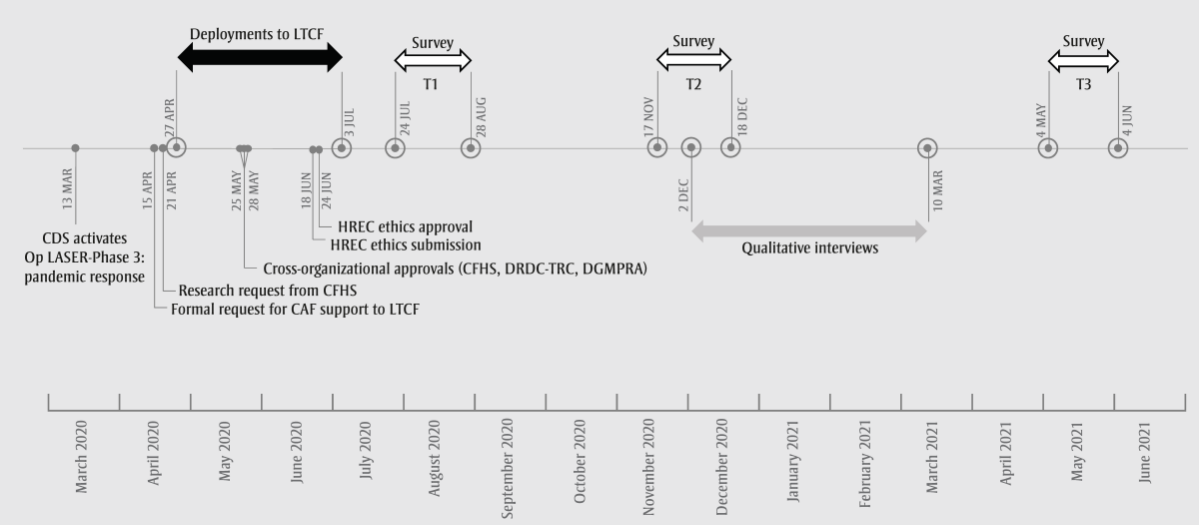
Timeline of Operation LASER long-term care facility (LTCF) deployment, approvals, and data collection. CAF: Canadian Armed Forces; CDS: Chief of Defence Staff; CFHS: Canadian Forces Health Services; DGMPRA: Director General Military Personnel Research and Analysis; DRDC-TRC: Defence Research and Development Canada–Toronto Research Centre; HREC: Human Research Ethics Committee; T1: time point 1; T2: time point 2; T3: time point 3.

This multimethod research initiative comprised 2 interrelated arms: a quantitative arm (ie, survey) and a qualitative arm (ie, interviews). The quantitative arm was a complete enumeration (ie, census) survey (ie, email invitations to participate were sent to all Operation LASER LTCF personnel) with self-report questionnaires administered remotely at 3 time points (referred to as *Survey*), allowing for cross-sectional and, for a subset of respondents, longitudinal analyses. The qualitative arm was a collection of in-depth, individual interviews with a focus on understanding the nuanced lived experiences of individuals taking part in the Operation LASER LTCF deployment (referred to as *Interviews*). Figure 1 shows the deployment, administrative approval, and data collection timelines (adapted with permission from the study by Fikretoglu et al [11]). No remuneration was provided for research participation.

### Survey

#### Participants

The target population for this survey was the entire cohort of CAF personnel deployed to Ontario and Québec LTCFs as part of Operation LASER LTCF. A list of the names and email addresses of 2595 CAF personnel, representing the deployed cohort at the time (June 26, 2020), was provided to the research team; military personnel on this list were eligible for participation in the study. There were no exclusion criteria.

#### Recruitment

An administrative manifest of all Operation LASER LTCF personnel deployed to Ontario and Québec as of June 26, 2020 (provided by CJOC), was used to establish the sample frame and disseminate survey invitations. The administrative list contained names, contact email addresses, military rank, and deployment location (ie, Québec or Ontario) for all CAF personnel deployed to Operation LASER LTCF. Considering that many Operation LASER LTCF personnel were reservists (who may not always have immediate access to internal Defence Wide Area Network [DWAN] email accounts), the list contained a mix of personal or DWAN addresses. As the survey invitation was sent to all individuals intended to be deployed to Operation LASER LTCF, survey sampling (eg, stratified random sampling) was not required or implemented. All personnel with valid contact information received an invitation email written in English and French at each data collection time point sent from the dedicated positional Operation LASER LTCF research project DWAN email address. Emails were individually and personally addressed (ie, rank and name) and briefly outlined the research objectives, methods, participant rights, risks, risk mitigation measures, benefits of the study, and relevant contact information. If individuals wished to participate, they were able to access the web-based questionnaire via individualized hyperlinks (directed to either the French or English version of the survey), which were accessible through the personal or DWAN email account. At each time point, reminder emails were sent to all participants who had not yet completed the survey for that specific time point. Each individual received a maximum of 2 email reminders per time point, spaced approximately 2 weeks apart.

#### Survey Administration Time Points

Survey invitations were sent on July 24, 2020; November 17, 2020; and May 4, 2021, for time point 1 (T1; 3 mo), time point 2 (T2; 6 mo), and time point 3 (T3; 12 mo), respectively. The respective data collection windows for the survey were July 24, 2020, to August 28, 2020; November 17, 2020, to December 18, 2020; and May 4, 2021, to June 4, 2021.

#### Measures

##### Overview

An overview of the measures included in the survey is presented in Table 1. To reduce respondent burden, branching logic was implemented where appropriate (ie, displaying relevant and hiding irrelevant sections of the survey based on previous responses). Similarly, not all sections were necessary to repeat at follow-up sessions (ie, demographic information and predeployment training). Most of the included measures had been validated in both French and English. Scales were adapted only in cases in which the item wording was not appropriate for the CAF or Operation LASER LTCF context (see the following sections).

**Table 1:** Operation LASER quantitative measure overview.

Assessment		Domain	Items, n	Baseline	Follow-up
Sociodemographic and military characteristics		Sociodemographics	7	✓	✓ (reduced)
Deployment characteristics^a^		Deployment characteristics	5	✓	✓
Predeployment training satisfaction^a^		Training	16	✓	—^b^
Prior CAF^c^ mental health or resilience training^a^		Training	8	✓	—
Personal protective equipment use^a^		Training	17	✓	—
Postdeployment satisfaction^a^		—	5	✓	—
C-PIQ^d^		COVID-19 impact	16	✓	—
**Stressful experiences**					
	Deployment stressors^a^	Stressful experiences	9	✓	✓ (reduced)
	MMD-LASER^a,^^e^	Moral distress	18	✓	✓ (expanded for T2 and T3)
**Mental health**					
	PHQ-9^f^	Depression symptoms	9	✓	✓
	GAD-7^g^	Anxiety symptoms	7	✓	✓
	PCL-5^h^	PTSD^i^ symptoms	21	✓	✓
	K-10^j^	General psychological distress	10	✓	✓
	MIOS^k^	Moral injury expression	40	✓	✓ (reduced to 14 items)
	Positive moral emotion outcomes^a^	Positive moral emotions	7	✓	✓
	Mental health service use	Mental health service use	3	—	✓
	Barriers to mental health service use	Barriers to mental health service use	23	—	✓ (expanded)
**Coping and social support**					
	COPE^l^ Inventory (brief version)	Coping styles	12	✓	✓
	Social support sources^a^	—	7	✓	✓
**Human Dimensions o** **f** **Operations**					
	Meaning scale^m^	Meaningfulness of work	3	✓	—
	Morale^m^	Motivation and enthusiasm	6	✓	—
	Relatedness^m^	Peer relatedness	7	✓	—
	Trust in teams^m^	Trust	9	✓	—
	Trust in leadership^m^	Trust	9	✓	—

^a^Scale or items created specifically for Operation LASER.

^b^Not collected.

^c^CAF: Canadian Armed Forces.

^d^C-PIQ: Complementary and Integrative Research Pandemic Impact Questionnaire.

^e^MMD-LASER: Measure of Moral Distress for Healthcare Professionals adapted to Operation LASER.

^f^PHQ-9: Patient Health Questionnaire–9.

^g^GAD-7: Generalized Anxiety Disorder Scale.

^h^PCL-5: Posttraumatic Stress Disorder Checklist for the Diagnostic and Statistical Manual of Mental Disorders, Fifth Edition.

^i^PTSD: posttraumatic stress disorder.

^j^K-10: Kessler Psychological Distress Scale.

^k^MIOS: Moral Injury Outcome Scale.

^l^COPE: Coping Orientation to Problems Experienced.

^m^Self-report assessment is a component of the Director General Military Personnel Research and Analysis (DGMPRA) Human Dimensions of Operations (HDO) data collection battery. Assessments were validated by the DGMPRA and designed specifically for administration to CAF personnel in HDO research.

##### Sociodemographic and Military Background

Information on age, gender, educational background, family status (ie, marital status, living arrangements, and dependents), military rank, length of military service, and component (ie, regular or reserve) was collected. These variables were selected based on previous research identifying them as potential predictors of psychological outcomes.

##### Operation LASER LTCF Roles and Duties

Participants were asked about the start and end dates of their Operation LASER LTCF deployment, military occupation (included only at T3), and the closest researcher-derived operational group they belonged to (Operation LASER 1, 2, 3, or 4; *other*; or *no role assigned*; the *no role assigned* option was available only at T3).

##### Predeployment Training and Postdeployment Decompression Satisfaction

Satisfaction with various aspects of predeployment training (ie, both for deployment duties and deployment psychological reactions) was assessed using 16 items that asked participants about their agreement with the following statement—*I am satisfied with my training for:*—for each of the 16 training components on a 7-point scale (ie, 1=*Strongly disagree*; 7=*Strongly agree*). A similar set of 5 questions assessed satisfaction with postdeployment training using an identical response format.

##### Prior CAF Mental Health or Resilience Training Questions

Exposure to and perceived utility of prior resilience training was assessed using items adapted from the 2013 Canadian Forces Mental Health Survey (CFMHS), a cross-sectional epidemiological survey conducted by Statistics Canada on behalf of the DND [27]. Items assessing the perceived utility of the training were adapted to be anchored to the Operation LASER LTCF context.

##### Coping

Various adaptive coping strategies were assessed using 16 items carefully selected from the well-established Brief Coping Orientation to Problems Experienced [28] based on the maximum relevance for Operation LASER LTCF as determined a priori by subject matter experts (SMEs). The selected items assess active coping, positive reframing, acceptance, use of emotional support, use of instrumental support, denial, substance use, and self-blame coping strategies. The following subscales were excluded: humor, venting, behavioral distraction, religion, behavioral disengagement, and self-distraction.

##### Social Support

Social support from various sources, including family, friends, employers, medical staff, religious or spiritual advisors, strangers, and online support groups, was assessed using a matrix of 7 items adapted from multiple sources, including the 2013 CFMHS [27]. Participants indicated their agreement with the following statement—*I received support from:*—on a 7-point scale (ie, 1=*Strongly disagree*; 7=*Strongly agree*).

##### Exposure to General Deployment Stressors

Exposure and perceived stress in response to general deployment stressors were captured using 9 items drawn from prior Human Dimensions of Operations surveys and from recent CAF Operation LASER LTCF reports [3,4] (Multimedia Appendix 1). Respondents indicated how stressed they felt in response to each of the situations (at the time of exposure) based on a 5-point scale (0=*No stress*; 4=*Extreme stress*, in addition to *N/A*=no exposure). The items were related to logistic support, accommodations, problems with infection control, lack of equipment and medication problems (eg, PPE), lack of civilian personnel, communication problems, absenteeism by civilian employees, and leadership or management issues. Participants were directed to indicate their degree of stress when the stressor was present (ie, at the time of deployment). Following T1 and T2 data collection, 3 items were excluded at T3 (ie, *problems with infection control*, *lack of equipment and medication problems (eg, PPE)*, and *civilian employee’s absenteeism problem*) because of redundancy and overlap with moral distress items (see the following section).

##### Exposure to Morally Distressing Experiences in Operation LASER LTCF

To assess the potential for moral challenges and distress in Operation LASER LTCF, we adapted the Measure of Moral Distress for Healthcare Professionals (MMD-HP [29]) for the context of Operation LASER LTCF (the adapted version was named MMD-HP adapted to Operation LASER [MMD-LASER]; Multimedia Appendix 2). Accordingly, participants were asked to rate how distressed they currently felt according to a 4-point scale in response to 20 moral situations adapted from the MMD-HP and via input from Operation LASER LTCF SMEs (eg, leadership, chaplains, and reports from deployed personnel). Scale modifications included reducing the number of items to display only those situations that may be encountered during Operation LASER LTCF and only measuring the degree of distress to reduce respondent burden (the original MMD-HP scale captured both frequency and distress). Participants were instructed to rate their degree of moral distress on a 4-point scale (ie, 1*=No moral distress*; 4=*Extreme moral distress*, in addition to *N/A=I have not experienced this situation*) for each of the potentially morally distressing experiences. The English-language MMD-LASER and the translated French version were not psychometrically tested at the time of survey administration. However, considering the formative model of measurement for this type of scale, ensuring that the scale contains a breadth of relevant exposures through rigorous consultation with Operation LASER LTCF SMEs may be a better indicator of scale quality than common quantitative psychometrics (eg, factor analysis and indexes of model fit), which are more appropriate for reflective measurement models [30].

##### Meaning

Grounded in theoretical understanding of psychological empowerment in the workplace [31], participants rated the perceived meaning of their duties during the operation using 3 statements on a 5-point scale (ie, 1=*Strongly disagree*; 5=*Strongly agree*, eg, *the work I do is very important to me*).

##### Relatedness

Relatedness refers to the sense of belonging a person experiences with others in the workplace [32]. Such attachments lead to a sense of being supported and can reduce the adverse effects of workplace stressors on mental health. Participants were asked to assess the extent to which they felt connected to the people they worked with by rating their level of agreement with 7 statements pertaining to perceived social support using a 5-point scale (ie, 1=*Totally disagree*; 5=*Totally agree*, eg, *at work, I can talk with people about things that really matter to me*).

##### Trust in Teams and Trust in Leadership

Participants were asked to rate their trust in their peers or team (eg, *My teammates were capable of doing their job*) and immediate leaders (eg, *My leader performed his/her job well*) [33] each using 9 items rated on a 5-point scale (ie, 1=*Completely disagree*; 5=*Completely agree*).

##### Morale

Participants were asked to rate their level of agreement with 6 statements pertaining to their morale and enthusiasm for accomplishing the objectives of Operation LASER LTCF using a 5-point scale (ie, 1=*Very low*; 5=*Very high*, eg, [Please rate] *your level of motivation*) [34].

##### Psychological Distress

Psychological distress was assessed using a self-report measure, the Kessler Psychological Distress Scale (K-10) [35]. The K-10 is a 10-item questionnaire assessing nervousness, agitation, fatigue, and negative affect. Good internal consistency (Cronbach α=.89-.92) and construct validity of the K-10 have been established in general population and military samples [35,36]. The French version of the K-10 has also shown good reliability (Cronbach α=.84) and validity [37].

##### Anxiety

The Generalized Anxiety Disorder Scale (GAD-7) [38] was used as a measure of anxiety. The GAD-7 is a 1-factor, 7-item self-report questionnaire that has demonstrated good internal consistency (Cronbach α=.89) and validity in both general population and primary care samples [39-41]. The French version of the GAD-7 has demonstrated good reliability (Cronbach α=.86) in a general population sample of French Canadians [42].

##### Depression

Severity of current depressive symptoms (ie, past 2 wk) was assessed using the Patient Health Questionnaire–9 (PHQ-9) [38]. The PHQ-9 is a validated and widely used brief, single-factor, 9-item self-report questionnaire with well-established reliability, validity, and sensitivity [43-45]. The French version of the PHQ-9 has demonstrated good internal consistency (Cronbach α=.83) [42] that is comparable with that of the original instrument.

##### Posttraumatic Stress Symptoms

Posttraumatic stress symptoms were assessed using the most commonly used self-report instrument for research purposes, the 20-item PTSD Checklist [46], which is based on the most recent conceptualization included in the Diagnostic and Statistical Manual of Mental Disorders [47]. A French-Canadian version has also undergone validation [48]. A new item was added at the end to determine whether the reported symptoms were related to or exacerbated by Operation LASER LTCF.

##### Moral Injury

Moral injury expression was assessed using the 14-item Moral Injury Outcome Scale (MIOS) self-report questionnaire [49]. To date, assessing moral injury has been a significant challenge because of the lack of well-conceptualized measures [50,51]. To address this gap, the MIOS was developed by an international consortium of leading experts in moral injury (across the United States, United Kingdom, Israel, Canada, and Australia). On the basis of the most distressing, potentially morally injurious event, participants were asked to indicate whether they would agree or disagree with statements that capture 6 domains: alterations in self-perception (eg, *my actions don’t fit with who I thought I was*), alterations in moral thinking (eg, *I have trouble seeing goodness in others*), social impacts (eg, *I feel rejected by people*), self-harming and self-sabotaging behaviors (eg, *I keep myself from having success*), emotional aftermath (eg, *I feel guilty*), and beliefs about life meaning and purpose (eg, *I lost a sense of meaning in life*). The MIOS has additional questions that further characterize the morally injurious event that the responses were based on. The version of the MIOS that was included in this study was a nonreduced 34-item version of the scale; scale validation on veteran samples has indicated a 14-item, 2-factor structure that will be used for scoring [49]. The French version of the MIOS has not yet been cross-validated.

##### Positive Reactions to Morally Challenging Situations

Haidt [52] has also suggested that, in addition to negative reactions such as moral injury, a number of positive reactions regarding oneself or others can result following the encounter of moral situations. As no validated measure exists for this construct, we created 7 items based on the description by Haidt [52], for instance, feeling pride in oneself or having faith in the goodness of humanity. Respondents were asked to indicate their agreement on a 5-point scale (ie, 0=*Strongly disagree*; 4=*Strongly agree*, eg, *I feel a sense of honour*) as to whether each of the statements characterized their reaction in the past month to their Operation LASER LTCF experience. This measure is currently not validated in English or French.

##### Posttraumatic Growth

Posttraumatic growth was assessed using 5 items drawn from the Posttraumatic Growth Inventory [53]. These items were selected to reflect the 5 factors assessed by the Posttraumatic Growth Inventory: stronger relationships, new possibilities, identification of personal strengths, spiritual change, and increased appreciation of life.

##### Perceived Need for Care

Participants were asked to indicate their subjective need for mental health care using a single, dichotomous self-report item adapted from the Canadian Community Health Survey [54] and the 2013 CFMHS [27] by Statistics Canada, which asked whether help for mental health problems (including emotions, mental health, or use of alcohol or drugs) was needed at any point since the start of Operation LASER LTCF.

##### Mental Health Service Use

A total of 3 items were taken from the 2013 CFMHS [27] to assess mental health service use at T2 and T3. The first item assessed access to formal and informal mental health support, and 2 follow-up items asked specifically about the Canadian Forces Member Assistance Program and web-based resources.

##### Barriers to Mental Health Service Use

Individuals may fail to seek care because of a denial of the need for treatment or because of perceived barriers to care [55]. Barriers to mental health care were measured using an 8-factor, self-report questionnaire developed by the Director General Military Personnel Research and Analysis (DGMPRA) in both French and English for a prior research project (Born, J, unpublished data, October 2021). Barriers to mental health care were explored using 29 items from the Operation LASER LTCF survey. A subset of 21 items was derived from the original 8-factor Barriers to Access to Care Evaluation, a 52-item scale that has demonstrated good internal consistency (Cronbach α=.86-.97) and validity (all factors predicted intention to seek care, and items are loaded in a theoretically logical manner) in a sample of CF H Svcs Gp personnel [24]. The abbreviated scale included 2 to 3 items per factor (8 factors in total: knowledge and ability to access care, organizational and social support, staffing and workload resources, concerns about privacy, alternative treatment options [eg, intention to self-treat], health care provider identity, conflict with career goals, and discomfort accessing care at work) selected from the larger Barriers to Access to Care Evaluation scale based on item factor loading scores, correlation of new factors with the original scale factor (*r*>0.90), and completeness of the factor’s content while maximizing both internal consistency and validity within the original sample of CF H Svcs Gp personnel. In addition, 3 new items were included in the survey to explore concerns about moral discomfort (eg, *Not comfortable discussing experiences where I felt I/others could/should have done more/acted differently*), 3 new items were added to explore revisiting trauma (eg, *I did not want to revisit stressful or traumatic experiences that would make me feel distressed*), 1 new item was added to explore quality of care (eg, *I did not have access to quality care*), and 1 item was added to explore past experiences (eg, *I have had a negative experience getting help in the past*) as barriers to mental health service use. Barriers to mental health service use were assessed at T2 and T3 using a 7-point scale rating agreement (ie, −3=*Strongly disagree* to +3=*Strongly agree*). The text of the chosen items was modified to distinguish between barriers experienced in the Operation LASER LTCF context for those with a perceived need for care and hypothetical barriers for those without a perceived need. At this point, neither the French nor the English version of the scale have been fully validated; nevertheless, it represents the only applicable measure currently available.

#### Survey Programing and Presentation

The survey was programmed in Snap Surveys (Snap Surveys Ltd [56]), a web-based survey platform that adheres to the Government of Canada’s quantitative research security policy. Individualized survey hyperlinks were given to each participant and were uniquely linked to each respondent’s email address; the web-based survey platform linked and stored contact information and a survey ID number separately from survey research data. This procedure preserved the deidentification of respondents while allowing for the linkage of their data across questionnaire administrations (ie, across different time points).

The survey began with a question that asked participants to select their preferred language (materials were available in both official languages of Canada [ie, English and French]). Once their preferred language was selected, an information page was displayed that reviewed the instructions, aims of the study, participant rights, and confidentiality. This was followed by questions that assessed demographics, prior training (Operation LASER LTCF–specific training for deployment duties and for psychological reactions, as well as prior resilience training), exposure to stressful experiences, moral injury, risk and resilience, and psychological health outcomes. The last section of the survey thanked respondents for their participation, informed them of the opportunity to volunteer for interviews (ie, interview study), and provided a list of available CAF mental health resources (from the CAF Directorate of Mental Health).

Review of survey content for relevance and order of presentation of various measures, dry-run completion for timings (to estimate respondent burden), and proofreading were conducted by internal research team members, CAF members either deployed to or familiar with Operation LASER LTCF, and carefully selected DND SMEs.

#### Data Analysis Plan

##### Survey Weight Generation

Depending on the patterns of nonresponse, certain demographic or military characteristics could be under- or overrepresented in the final sample. Estimates produced using the final sample without any weight adjustment would be representative of the sample itself but not necessarily of the source population. To reduce nonresponse bias and improve the representativeness of the study findings to the source population, we generated survey weights based on available sociodemographic and military characteristics of the source population (see the *Data Linkage* section). A total of 5 characteristics were available for the source population: age, gender, force type, rank, and province deployed. Given our limited sample size, we could not conduct poststratification using all 5 weighting factors. To maximize the effect of using weights to adjust for nonresponse bias, we combined poststratification and propensity score inverse weighting in generating our survey weights [57]. We first used propensity score inverse weighting with all 5 weighting factors included in a binary logistic regression model predicting response at each time point. Given the sample size, the interaction terms of the weighting factors were not included. For each respondent, a weight was created as the inverse of the propensity score of responding with the sum of the weights equal to the sample size of the source population. The second step was to poststratify weights from the first step based on age and gender information. A total of 4 cells were created, and weight adjustment was used for each participant according to which cell the participant fell into. For each cell, the weight adjustment factor was calculated as the ratio of the sum of the weights from the first step to the sample size of the source population in that cell. This step ensured that the weighted sample had the same distribution in terms of age and gender as the source population. The third step was to apply a weight adjustment to reflect the original sample size at each time point. This step served to avoid artificially inflating the sample size while preserving the representativeness of the source population. The last step was to give participants with missing information in the 5 weighting factors a weight of 1 (2.7% of the source population). This step allowed for keeping these participants in the data analysis in the absence of information by adjusting for their response bias. Analyses with the application of the generated survey weights are expected to provide estimates that are statistically adjusted to reflect key source population parameters, making the results generalizable to the source population. Using the same process, an alternative set of survey weights was generated based on a sample (981/1088, 90.17% at T1; 445/582, 76.5% at T2; and 403/497, 81.1% at T3) where the definition of a partial responder had a higher threshold for completion (ie, at least 1 section answered that related to a study outcome, defined as any of the assessments following Operation LASER LTCF Roles and Duties [see the *Measures* section for survey order]). This approach treated early *break-off* participants as unit nonresponders, similar to previously published guidelines [58]. A sensitivity analysis will be conducted to assess the effect of using different samples for weight generation on the study findings.

##### Data Linkage

To compute sample weights for source population representativeness, additional demographic variables (ie, date of birth, gender, force type, years of service, Operation LASER LTCF role, time in mission, occupation, unit name, deployment start date, and deployment end date) were required. In early March 2021, a Military ad hoc request was submitted to the office of the Assistant Deputy Minister (Information Management) to provide these additional variables, as well as variables for data linkage (ie, service number [SN], first name and last name), for all CAF personnel who were deployed on Operation LASER LTCF between April 1, 2020, and August 30, 2020. Typically, a unique identifier variable (ie, SN) would be used to link datasets; however, although SNs were provided in the ad hoc administrative file for all members, SNs were missing for 32% of members from the original administrative list. To confidently match as many members from the administrative list as possible, a sequence of automated and manual matching iterations was completed on the available identifiers, resulting in a total of 2536 (97.77% of the sample linked to administrative data) confirmed matches of 2595 possible matches. This allowed for a fairly accurate computation of sample weights for further weighted analyses.

##### Careless Responses

Checks for careless responders at each time point were conducted using a combination of post hoc methods following recommendations by Meade and Craig [59]. The *careless* R package (R Foundation for Statistical Computing) [60] was used to assist in computing carelessness indexes. Specifically, indexes included longest identical sequence, average identical sequence, intraindividual response variability split across 5 equal sections of the survey (starting with MMD-LASER, see the following rationale), psychometric synonym index, and Mahalanobis distance (for more information, see the study by Meade and Craig [59]). We avoided calculating carelessness indexes in sections where conditional question presentation (ie, display logic) was common (eg, training satisfaction and deployment details); as such, all scales including and following the MMD-LASER were used in the calculation of the indexes. These indexes were mapped to response times to identify individuals with unusual response patterns (ie, very short completion times in combination with significant outlier values [–2.5-+2.5 SD] on 1 or more of the carelessness indexes). The responses of these individuals were then manually checked by a research team member to confirm their response patterns and identify substantive evidence for careless responses. A conservative approach of not excluding any participants was taken in light of the nature of the sample (ie, largely psychologically healthy) and the measures (ie, largely clinical) that were included in calculating the indexes; a floor effect on these scales may create false positives for certain indexes, such as the longest and average identical sequence.

##### Statistical Analysis

Depending on the analytical objectives, the data will be analyzed cross-sectionally or longitudinally. Population-averaged approaches, such as generalized estimation equations, will be chosen for objectives that aim to assess population means. For objectives that aim to identify risk factors at the individual level, individual-specific approaches such as hierarchy regressions will be chosen. Sample weights will be used in all analyses to improve the representativeness of the study estimates for the source population. Considering the response rate and uncertainty of the reason for nonresponse, both weighted and unweighted estimates will be presented, and their discrepancies will be discussed. In addition, weights will be applied to all longitudinal analyses to account for bias owing to potential differential attrition. A 95% CI for estimates will be provided wherever possible.

No individual-level data or tables (eg, cross-tabulations) that may identify individuals will be produced. Cell sizes of <5 will not be reported. Descriptive statistics (eg, mean, SD, and frequencies) summarized at the group level will be used to capture key outcomes cross-sectionally (ie, at each of the 3 time points). Inferential statistics (eg, ANOVA, regression, and correlation) using T1 data will be conducted to examine the relationships between the risk and resilience factors and the key outcomes, as well as the differences that may exist between subgroups of the cohort (eg, differences in trust or relatedness between ranks).

Path analysis and structural equation modeling using T1 data will be used to analyze potential conceptual models. Predictive models (eg, linear and binary logistic regressions) using T1 data to capture changes over time will be used to assess associations between risk and resilience factors and the key outcomes. SPSS Statistics (version 23; IBM Corp), R (version 4.0.5), RStudio (version 1.4; Posit, PBC), and SAS (version 9.4; SAS Institute) will be used for all data management and analyses.

### Interviews

#### Participants and Recruitment

The target population for the interview component of the project was the entire cohort of CAF personnel deployed to Ontario and Québec LTCFs as part of Operation LASER LTCF. Considerations regarding project resourcing and data saturation limited the maximum sample size of the qualitative interviews to approximately 50. Interviews were conducted in the participants’ choice of French or English. There were no exclusion criteria.

On December 2, 2020, an email call for participants (written in English and French) was sent from the dedicated positional Operation LASER LTCF research project DWAN email address to the entire cohort of CAF personnel deployed to LTCFs (N=2595) to invite them to participate in a confidential interview with the research team. Similar to the survey study, emails were individually and personally addressed (ie, rank and name) and briefly outlined the study objectives, methods, participant rights, risks, risk mitigation measures, benefits of the study, and relevant contact information. Interested personnel were instructed to reply to the email to contact a member of the research team to obtain more information about the study or schedule an interview. The Operation LASER LTCF study email address was monitored by 2 members of the research team. Responses to prospective participants were provided in the same language as that used in their email inquiries.

In an effort to select a representative sample, from the pool of personnel who expressed interest in taking part in the interview study, participants were selected based on their Operation LASER LTCF role, rank, gender, component, province of deployment, and marital status. At the time of recruitment for the interview study, the research team did not have access to the ad hoc report, which provided demographic information for the entire population of Operation LASER LTCF personnel. As a result, participants were selected to ensure that the overall demographic breakdown of the interview study sample reflected that reported by respondents in the T1 survey. This sampling method yielded relatively representative proportions of the overall population (Table S1 in Multimedia Appendix 3) and ensured relative representation from subpopulations with a range of responsibilities and experiences. A member of the research team emailed the selected participants to schedule a 1-hour interview based on participants’ availability and language of choice. A study information sheet was attached to the scheduling email to provide further details.

#### Interview Process

The interviews were conducted using Teams (Microsoft Corp), a DND-approved communication platform that allows for web-based synchronic audio and video discussions. Participants had the choice to join the web-based discussion, with their video turned on or off, or to dial in by telephone. At the outset of each scheduled interview, the research goals, as well as participant rights and protections stated in the information sheet, were outlined. Verbal consent to participate in the interview and to have their interview audio recorded (to assist the researchers with note taking) was obtained from each participant. Each interview was conducted in the official language of the respondents’ choice. For consistency, one interviewer conducted most of the interviews while a second interviewer took notes, managed the audio recorder, and asked follow-up questions when applicable. Each interview was scheduled for 1 hour; however, an additional 30 minutes was scheduled between interview sessions in the event that a participant desired more time to share the details of their unique experiences. The duration of the interview was ultimately up to the participant. A semistructured interview approach was used, in which participants were guided by specific questions that aimed to explore their pre-, peri-, and postdeployment experiences. All interviews were conducted between December 2, 2020, and March 30, 2021.

#### Interview Content

The discussion domains are summarized in Multimedia Appendix 4 and fall under the general categories of (1) demographic and background variables, (2) predeployment experiences, (3) perideployment experiences, and (4) postdeployment experiences. These areas allowed for a nuanced view of demographic and background factors that may affect the Operation LASER LTCF experience [7,13,14]; Operation LASER LTCF–specific risk and protective factors, such as individual exposure and psychological reactions to stressful Operation LASER LTCF events; coping styles; and experiences related to moral challenges, moral emotions, moral distress [61], and potential moral injury [12,17,25,52,62]. Participants were also asked about group cohesion and leadership factors that may have affected their experience as past research has demonstrated that social-, unit-, leader-, and organizational-level factors may also affect team dynamics (Michaud K, unpublished data, December 2021). Participants were also asked about mental health service use and barriers to help seeking among CAF members [22-24]. Finally, participants were queried on any lessons learned that could be shared with military leadership to inform preparation and support for future missions of the same nature. Although these predefined topics guided the discussions, participants were free to raise additional issues to discuss in the interviews.

#### Analytical Approach to Qualitative Data

The audio recording of each discussion conducted in either French or English was transcribed verbatim into a text file. French transcriptions were translated into English for data analysis purposes, and the English transcriptions were uploaded to the NVivo qualitative analysis software (QSR International). Each discussion was first analyzed by the research team (who were also the interviewers) to identify the main topics addressed (called nodes) throughout the data. Once this coding scheme was complete, NVivo was used to generate a word search to discover trends throughout the discussions. For example, the program identified how often certain words were used, especially with regard to military training, military experience, and postdeployment particularities. By choosing keywords or nodes, the software presented each of the references related to a specific node, which allowed for the comparison of the results across different keywords or nodes and the detection of trends throughout the discussions.

Each interview transcript was manually coded according to the following contexts: before deployment (eg, the military role in Operation LASER LTCF, the predeployment training according to that role, and the domestic operation dimension and expectations), during deployment (eg, location, duration, role, experience, competence, chain of command, communication with civilian staff and with other military personnel, and sanitary measures), and after deployment (eg, quarantine, postdeployment training, return home and family, and willingness to deploy to another similar domestic or overseas operation). Further details on the qualitative methodology will be reported in follow-up manuscripts specific to each analysis and research question.

### Ethical Considerations

The CAF Surgeon General and Chief of Military Personnel were the sponsors of this research, and CJOC approved access to Operation LASER LTCF personnel. This survey was conducted jointly by Defence Research and Development Canada, DGMPRA, CF H Svcs Gp, and the Royal Military College of Canada. It was reviewed and approved by the Defence Research and Development Canada Human Research Ethics Committee (approval HREC 2020-026 and 2020-040) and coordinated by the DGMPRA Social Science Research Review Board in accordance with Defence Administrative Orders and Directives 5062-0 and 5062-1 (coordination 1897/20N). The questions asked in this research fell under the current Treasury Board of Canada Secretariat definition of public opinion research. Thus, public opinion research approval from the Deputy Minister was obtained before the survey launch (ie, July 21, 2020). Although CJOC approved and facilitated this study by coordinating the provision of contact information of potential participants, neither the research sponsors nor CJOC were directly involved in participant recruitment for any phase or aspect of the study, nor did they know the identities of the individuals who elected to participate. An unsigned consent procedure was used, consistent with the methodology used in previous surveys administered by DGMPRA. All data used for analysis were deidentified. No participant remuneration was provided.

## Results

### Survey

#### Cohort and Survey Frame Size

A total of 47, 12, and 13 invitations were not delivered because of automated email bounce messages for T1, T2, and T3, respectively (representing 13/2595, 0.5% to 47/2595, 1.81% of all eligible personnel). Both the Ontario and Québec points of contact were asked for updated email addresses for members whose email addresses were invalid. An updated Québec list was received soon after the initial T1 invitation email was distributed; thus, members with an updated email address were sent the initial T1 survey invitation and all subsequent reminders. For Ontario members, an updated list was received before the distribution of the initial T2 survey invitation, which accounts for the decrease in undeliverable emails between T1 and T2. Although most invalid email addresses were personal, a few emails to DWAN accounts also bounced; the reason for this issue was unknown. A total of 0.31% (8/2595) of the personnel asked to be removed from the distribution list after either the T2 or interview study emails were sent out; an additional member asked to be removed after the initial T3 email was sent. Ultimately, survey invitations were sent to 2595 members at T1 and T2 and 2587 individuals at T3.

#### Participation Metrics and Sample Characteristics

##### Participation Across Time Points

**Figure 2:**
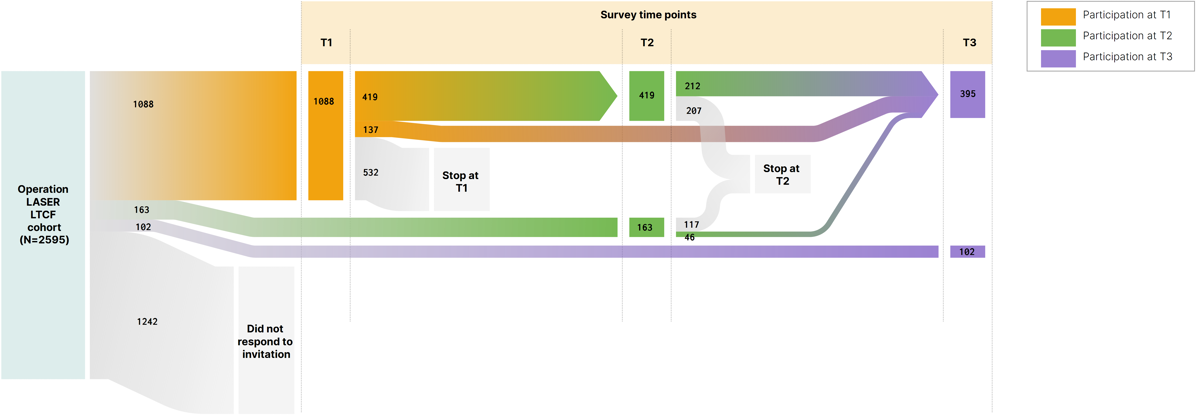
Survey participation flow across data collection time points. LTCF: long-term care facility; T1: time point 1; T2: time point 2; T3: time point 3.

The participation flow across the 3 data collection time points is shown in Figure 2. Overall, 1088, 582, and 497 individuals responded in T1, T2, and T3, respectively (see Tables 2-4 for unweighted and weighted sample characteristics). An individual was classified as a responder if they completed at least one question in the first section of the survey (ie, demographics and Operation LASER LTCF role). Relative to the total cohort eligible for participation (N=2595), this represented a participation rate of 41.93% (1088/2595), 22.43% (582/2595), and 19.15% (497/2595) for T1, T2, and T3, respectively. Close to half (532/1088, 48.9%) of the T1 participants did not return for follow-up at T2 and T3. Of those 1088 who participated in T1, a total of 419 (38.5%) had data at T2, a total of 212 (19.5%) had data for all 3 time points, and 137 (12.6%) had data only for T1 and T3. Not all individuals who participated in the follow-up time points (ie, T2 and T3) participated in T1. At T2, a total of 28% (163/582) of all participants took part for the first time. More than a quarter of these T2 first-time participants (46/163, 28.2%) continued to T3. T3 had 20.5% (102/497) of first-time participants.

**Table 2:** Unweighted and weighted frequencies and percentages of sample characteristics at time 1.

Characteristics				Unweighted results (n=1088), n (%)^a^		Weighted results (n=1088.02), n (%; 95% CI)^a^
**Demographics**						
	**Age group (y)**					
		17-29	558 (56.08)		650.57 (65.29; 62.40-68.17)	
		30-39	295 (29.65)		234.64 (23.55; 21.05-26.04)	
		40-58	142 (14.27)		111.24 (11.16; 9.37-12.95)	
	**Gender**					
		Female	280 (26.02)		205.55 (19.11; 16.97-21.25)	
		Male	796 (73.98)		869.85 (80.89; 78.75-83.03)	
	**Marital status**					
		Single	610 (56.12)		672.14 (61.81; 58.90-64.72)	
		Separated, divorced, or widowed	46 (42.3)		38.18 (3.51; 2.48-4.54)	
		Married or common-law partnership	431 (39.65)		377.1 (34.68; 31.84-37.51)	
	**Home situation**					
		Dependents	307 (28.37)		260 (24.03; 21.55-26.51)	
		No dependents	775 (71.63)		822 (75.97; 73.49-78.45)	
**Military factors**						
	**Force type**					
		Regular force	511 (47.67)		452.51 (42.24; 39.23-45.25)	
		Reserve force	561 (52.33)		618.68 (57.76; 54.75-60.77)	
	**Rank**					
		NCM^b^	862 (79.45)		909.71 (83.81; 81.77-85.86)	
		Officer	223 (20.55)		175.69 (16.19; 14.14-18.23)	
**Operation LASER**						
	**Province**					
		Ontario	338 (31.98)		351.25 (33.31; 30.34-36.28)	
		Québec	698 (66.04)		683.99 (64.87; 61.87-67.87)	
		Both	16 (1.51)		14.77 (1.4; 0.70-2.11)	
		Other	2 (0.19)		2.03 (0.19; 0-0.47)	
		Other but could be both	3 (0.28)		2.32 (0.22; 0-0.47)	
	**Role**					
		Operation LASER 1	382 (35.87)		351.03 (33.08; 30.21-35.95)	
		Operation LASER 2	210 (19.72)		241.55 (22.76; 20.05-25.48)	
		Operation LASER 3	71 (6.67)		78.46 (7.39; 5.70-9.09)	
		Support roles	344 (32.3)		328.76 (30.98; 28.14-33.82)	
		Other	45 (4.23^c^)		47.82 (4.51; 3.18-5.83)	
		None	13 (1.22)		13.54 (1.28; 0.56-1.99)	

^a^Raw n may not always be equal to the total sample size due to data missingness. Percentages are based only on data that is present.

^b^NCM: noncommissioned member.

**Table 3:** Unweighted and weighted frequencies and percentages of sample characteristics at time 2.

Characteristics				Unweighted results (n=582), n (%)^a^		Weighted results (n=582.01), n (%;95% CI)^a^
**Demographics**						
	**Age group (y)**					
		17-29	263 (50.87)		327.51 (63.62; 59.53-67.71)	
		30-39	161 (31.14)		120.38 (23.38; 19.93-26.84)	
		40-58	93 (17.99)		66.92 (13; 10.39-15.61)	
	**Gender**					
		Female	142 (25.54)		107.69 (19.47; 16.35-22.59)	
		Male	414 (74.46)		445.47 (80.53; 77.41-83.65)	
	**Marital status**					
		Single	292 (52.05)		336.64 (60.21; 56.06-64.35)	
		Separated, divorced, or widowed	28 (4.99)		24.66 (4.41; 2.73-6.09)	
		Married or common-law partnership	241 (42.96)		197.85 (35.38; 31.39-39.38)	
	**Home situation**					
		Dependents	182 (32.62)		148.00 (26.6; 22.98-30.22)	
		No dependents	376 (67.38)		408.00 (73.4; 69.78-77.02)	
**Military factors**						
	**Force type**					
		Regular force	273 (49.28)		235.65 (42.8; 38.53-47.07)	
		Reserve force	281 (50.72)		314.96 (57.2; 52.93-61.48)	
	**Rank**					
		NCM^b^	424 (75.58)		464.03 (82.99; 80.19-85.79)	
		Officer	137 (24.42)		95.11 (17.01; 14.21-19.81)	
**Ope** **ration** **LAS** **ER**						
	**Province**					
		Ontario	176 (31.43)		182.24 (32.67; 28.53-36.80)	
		Québec	367 (65.54)		360.16 (64.56; 60.36-68.77)	
		Both	13 (2.32)		11.78 (2.11; 0.91-3.31)	
		Other	1 (0.18)		1.46 (0.26; 0-0.78)	
		Other but could be both	3 (0.54)		2.22 (0.4; 0-0.86)	
	**Role**					
		Operation LASER 1	206 (36.79)		192.42 (34.49; 30.40-38.59)	
		Operation LASER 2	110 (19.64)		132.41 (23.74; 19.83-27.64)	
		Operation LASER 3	35 (6.25)		40.27 (7.22; 4.83-9.61)	
		Support roles	183 (32.68)		164.20 (29.43; 25.57-33.30)	
		Other	15 (2.68)		17.16 (3.08; 1.48-4.67)	
		None	11 (1.96)		11.39 (2.04; 0.77-3.31)	

^a^Raw n may not always be equal to the total sample size due to data missingness. Percentages are based only on data that is present.

^b^NCM: noncommissioned member.

**Table 4:** Unweighted and weighted frequencies and percentages of sample characteristics at time 3.

Characteristics				Unweighted results (n=497), n (%)^a^		Weighted results (n=497.00), n (%;95% CI)^a^
**Demographics**						
	**Age group (y)**					
		17-29	208 (46.12)		282.60 (63.08; 58.67-67.48)	
		30-39	149 (33.04)		101.76 (22.71; 19.14-26.28)	
		40-58	94 (20.84)		63.66 (14.21; 11.35-17.06)	
	**Gender**					
		Female	116 (23.82)		89.94 (18.54; 15.17-21.91)	
		Male	371 (76.18)		395.19 (81.46; 78.09-84.83)	
	**Marital status**					
		Single	229 (46.83)		274.53 (56.42; 51.79-61.04)	
		Separated, divorced, or widowed	26 (5.32)		20.25 (4.16; 2.44-5.89)	
		Married or common-law partnership	234 (47.85)		191.83 (39.42; 34.92-43.93)	
	**Home situation**					
		Dependents	173 (35.45)		136.00 (27.88; 23.92-31.84)	
		No dependents	315 (64.55)		351.00 (72.12; 68.16-76.08)	
**Military factors**						
	**Force type**					
		Regular force	251 (51.54)		213.61 (44.06; 39.39-48.73)	
		Reserve force	236 (48.46)		271.18 (55.94; 51.27-60.61)	
	**Rank**					
		NCM^b^	376 (76.89)		404.08 (83.01; 79.86-86.15)	
		Officer	113 (23.11)		82.73 (16.99; 13.85-20.14)	
**Operation LASER**						
	**Province**					
		Ontario	171 (35.19)		163.01 (33.64; 29.18-38.11)	
		Québec	301 (61.93)		310.13 (64; 59.47-68.54)	
		Both	12 (2.47)		10.20 (2.1; 0.84-3.37)	
		Other	0 (0)		0 (0; 0)	
		Other but could be both	2 (0.41)		1.23 (0.25; 0-0.60)	
	**Role**					
		Operation LASER 1	178 (36.85)		159.74 (33.35; 28.93-37.77)	
		Operation LASER 2	92 (19.05)		116.18 (24.26; 19.86-28.65)	
		Operation LASER 3	22 (4.55)		27.08 (5.65; 3.29-8.02)	
		Support roles	165 (34.16)		149.78 (31.27; 26.92-35.62)	
		Other	7 (1.45)		6.21 (1.3; 0.26-2.33)	
		None	19 (3.93)		19.99 (4.17; 2.24-6.11)	

^a^Raw n may not always be equal to the total sample size due to data missingness. Percentages are based only on data that is present.

^b^NCM: noncommissioned member.

##### Completion Time

Multimedia Appendix 5 describes the quantiles of completion time for individuals who completed the survey (ie, reached and submitted the final page). Survey duration for partial completers (ie, completed at least one question in the first section of the survey and left the survey before reaching the final page) was not available because of the manner in which time stamps were established by the survey platform (ie, only recorded at the start of the survey and once participants submitted the final page). After the removal of unreasonably long durations (ie, >500 min, likely because of participants completing the survey in multiple sittings), the median completion time for T1, T2, and T3 was 35.2 (IQR 26.3) minutes, 22.1 (IQR 16.0) minutes, and 25.9 (IQR 19.6) minutes, respectively. As expected, T1 had the longest completion duration because of the number of scales and items in the survey. Although T2 and T3 were very similar in content, additional questions were included in T3 that were not present in T2 (eg, additional items in the MMD-LASER). In reviewing the completion dates of the unusually long survey durations, it was evident that respondents returned to the survey on a different day to complete it as it aligned with the dates when reminder emails were sent. This pattern indicated that reminder emails were effective in encouraging a portion of partial completers to complete the survey at a later date (representing approximately 5%-10% of respondents at each time point).

##### Attrition Across Survey Sections

**Figure 3:**
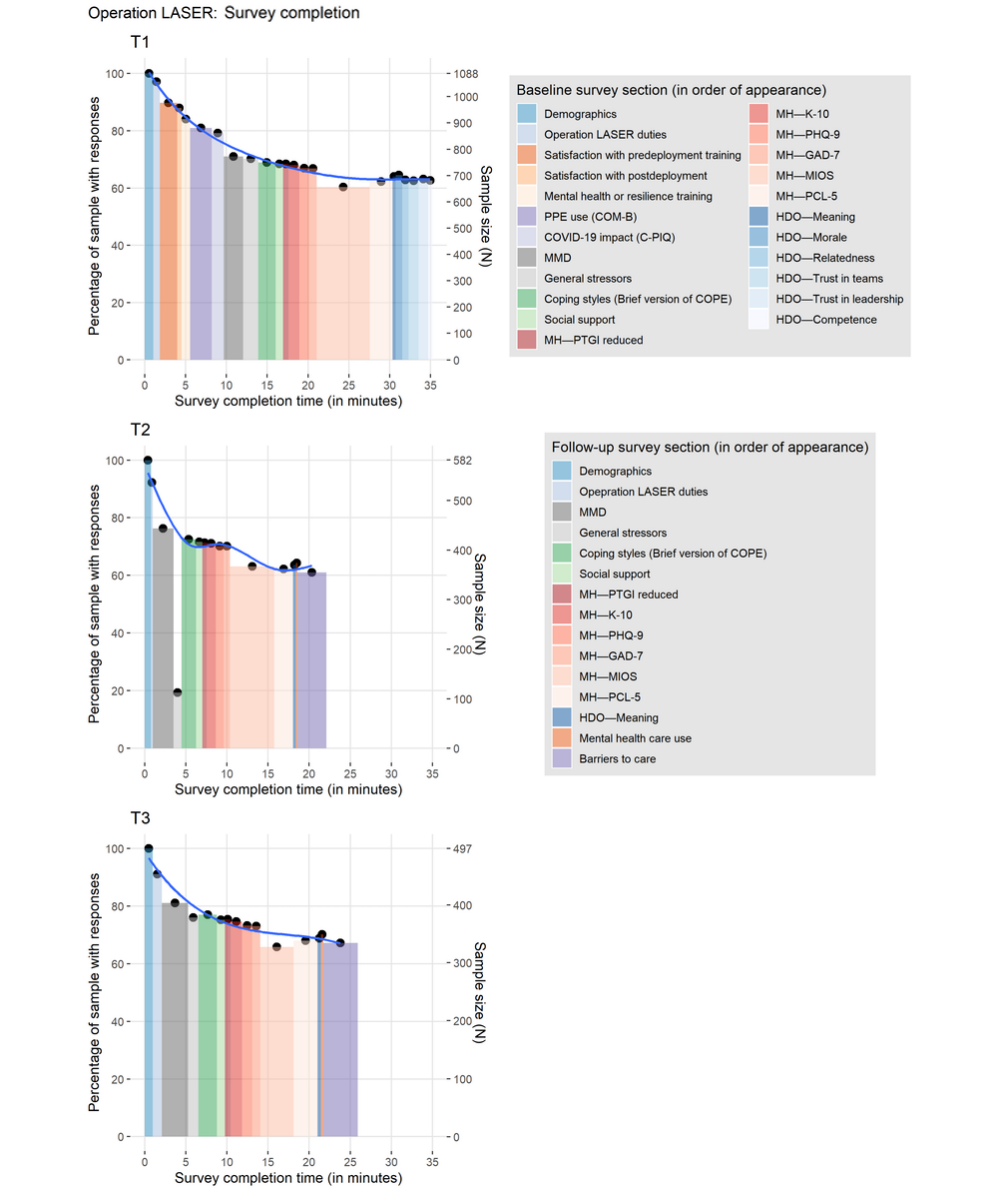
Participant attrition across survey sections. C-PIQ: Complementary and Integrative Research Pandemic Impact Questionnaire; COM-B: Capability, Opportunity, and Motivation–Behavior; COPE: Coping Orientation to Problems Experienced; GAD-7: Generalized Anxiety Disorder Scale; HDO: Human Dimensions of Operations; K-10: Kessler Psychological Distress Scale; MH: mental health; MIOS: Moral Injury Outcome Scale; MMD: Measure of Moral Distress; PCL-5: Posttraumatic Stress Disorder Checklist for the Diagnostic and Statistical Manual of Mental Disorders, Fifth Edition; PHQ-9: Patient Health Questionnaire–9; PPE: personal protective equipment; PTGI: Posttraumatic Growth Inventory; T1: time point 1; T2: time point 2; T3: time point 3.

Common to many web-based surveys, not all participants in our study who started the survey completed it (see Figure 3 for attrition across survey sections). Survey completion at each time point was approximately 60% to 65% (relative to the number of individuals who completed the first section of the survey at each time point). Attrition across survey sections was largely a function of time spent in the survey, where approximately 80% of participants were retained at the 5-minute mark and 70% of participants were retained at the 10-minute mark.

### Interview

A total of 208 members responded to our request for participants for the interview study, representing approximately 8.02% (208/2595) of the eligible cohort. Of the 208 participants who responded, 185 (88.9%) expressed interest in participating, 14 (6.7%) requested more information, and 9 (4.3%) declined the invitation to participate or requested to have their name removed from the distribution list for future Operation LASER LTCF research invitations. In total, 53 interviews were conducted (n=23, 43% in French, n=27, 51% in English, and n=3, 6% in both languages). Multimedia Appendix 4 shows the characteristics of the interview participants.

All participants except 1 (52/53, 98%) consented to having their discussion audio recorded. In the case of the individual who declined audio recording, the investigator leading the discussion took detailed notes. The interviews ranged in duration from 34 to 98 minutes, lasting approximately 70 minutes on average. All discussions were transcribed verbatim (with the exception of the one interview in which the participant did not consent to audio recording). The number of words per transcript ranged from 3114 to 19,340, with a mean of 9779 words. Most of the personnel who participated in the interview study also completed the survey (51/53, 96%).

## Discussion

This paper documents the methodology and implementation of a longitudinal study that aims to explore the psychological impact and mental health outcomes of CAF personnel deployed domestically to support civilian health care in LTCFs during the COVID-19 pandemic (as part of Operation LASER). Owing to the moral nature of the stressors encountered by personnel deployed to LTCFs and the insufficiently researched link between morally injurious experiences and mental health outcomes, it was important to understand their impact on mental health and well-being. The research objectives included identifying and quantifying individual, group, and organizational risk and resilience factors associated with these mental health outcomes; gaining an in-depth understanding of the personnel’s lived experiences during deployment; and extracting lessons to inform decisions for similar future deployments.

Overall, the study rollout and data collection were successfully executed despite the complexity of the study design. The participant response rates, ranging from 19.15% (497/2595) to 41.93% (1088/2595) depending on the time point, are typical and consistent with those of other studies [63], including those in military populations [64-66]. Of those who started the survey, most completed it in its entirety. The likelihood of survey dropout was a function of survey length, with the largest dropout (approximately 20%) occurring within the first 5 minutes. This attrition across survey sections is comparable with that of other web-based surveys, suggesting that, as the time requirement for a survey increases, the completion rate decreases [67,68].

Among the several objectives of this study, a critical gap that this research intended to address lay in the area of moral injury. Military personnel often face situations that may be perceived to transgress deeply held moral beliefs and values; this includes through acts of commission of, omission of, learning about, or being a victim of betrayal [69,70]. Such experiences can lead to moral injury, causing intense feelings of guilt, shame, spiritual conflict, social alterations, and changes in moral thinking, which are distinct from PTSD but can coexist with it [71,72]. Understanding the nuances of moral injury (ie, its risk factors and mechanism of development) can help further differentiate it from PTSD and develop more tailored mental health interventions. These interventions can range from predeployment training programs that prepare military personnel for the moral complexities of operations to therapeutic approaches that can assist in the healing process for those experiencing moral injury. Finally, the identification and recognition of the scope and impact of moral injury can help foster a military culture that is more aware of and responsive to the moral and ethical challenges of military service, particularly when emerging research highlights the protective role of organizational support and sound leadership against the development of distress in the aftermath of exposure to potentially morally injurious events [19,73]. Addressing these gaps is not only a priority for military personnel [50,74] but also relevant to other high-stress occupational settings where individuals may be presented with moral conflicts in high-stakes environments (ie, first responders, health care workers, and the legal system) [75-77].

Despite the longitudinal and mixed methods nature of the study, the substantial effort made to optimize the study design mitigates bias (eg, attrition and nonresponse bias and careless responding). This study is not without limitations. Specifically, the lack of a time point preceding deployment limits the ability to draw strong causal inferences linked to the experiences during deployment. Data for this study were collected using web-based self-reports, which may carry the inherent bias associated with most self-reports, including measurement error, human error, response bias (eg, social desirability), memory recall bias, interpretation challenges, and lack of verifiability. Inherent to longitudinal surveys, attrition bias and panel conditioning may have been a factor. Finally, the 3–time point longitudinal design, although superior to trend study designs, has limited time resolution and restrains the granularity of temporal changes in the key outcomes captured in this survey.

The quantitative and qualitative data generated through this research provide an opportunity to characterize the well-being and mental health of Operation LASER LTCF personnel over the year after this deployment, identify general and Operation LASER LTCF–specific risk and protective factors, and inform preparation and interventions for future missions.

## Appendix

Multimedia Appendix 1 General deployment stressor items.

Multimedia Appendix 2 Items on the Measure of Moral Distress for Healthcare Professionals adapted to Operation LASER.

Multimedia Appendix 3 Interview study discussion domains.

Multimedia Appendix 4 Characteristics of the interview study sample.

Multimedia Appendix 5 Distribution of completion times for time points 1, 2, and 3.

## Abbreviations

CAF: Canadian Armed Forces
CF H Svcs Gp: Canadian Forces Health Services Group
CFMHS: Canadian Forces Mental Health Survey
CJOC: Canadian Joint Operations Command
DGMPRA: Director General Military Personnel Research and Analysis
DND: Department of National Defence
DWAN: Defence Wide Area Network
GAD-7: Generalized Anxiety Disorder Scale
K-10: Kessler Psychological Distress Scale
LTCF: long-term care facility
MIOS: Moral Injury Outcome Scale
MMD-HP: Measure of Moral Distress for Healthcare Professionals
MMD-LASER: Measure of Moral Distress for Healthcare Professionals adapted to Operation LASER
PHQ-9: Patient Health Questionnaire–9
PPE: personal protective equipment
PTSD: posttraumatic stress disorder
SME: subject matter expert
SN: service number
T1: time point 1
T2: time point 2
T3: time point 3

## Data Availability

The data sets generated and analyzed during this study are not publicly available. Access to research data is restricted and governed by Department of National Defence and Director General Military Personnel Research and Analysis policies.
