# Supplementary material for: Moral Distress, Mental Health, and Risk and Resilience Factors Among Military Personnel Deployed to Long-Term Care Facilities During the COVID-19 Pandemic: Research Protocol and Participation Metrics
Source: JMIR Res Protoc. 2023 Nov 6;12:e44299. doi: 10.2196/44299 (PMC10629501; doi:10.2196/44299)
Supplement: Multimedia Appendix 1 [file resprot_v12i1e44299_app1.docx]

## Table S1. Items included in the General Deployment Stressors Scale.

1. Your logistic support
2. Your accommodations
3. Lack of civilian personnel
4. Communication problems
5. Leadership or management issues
6. Being unsure of when your support to Op LASER will end
7. Problems with infection control**
8. Civilian employee's absenteeism problem**
9. Lack of equipment and medication problems (e.g., PPE)**

** Removed at T3
