# Supplementary material for: Moral Distress, Mental Health, and Risk and Resilience Factors Among Military Personnel Deployed to Long-Term Care Facilities During the COVID-19 Pandemic: Research Protocol and Participation Metrics
Source: JMIR Res Protoc. 2023 Nov 6;12:e44299. doi: 10.2196/44299 (PMC10629501; doi:10.2196/44299)
Supplement: Multimedia Appendix 2 [file resprot_v12i1e44299_app2.docx]

## Multimedia Appendix 2

## Table S2. Items included in the novel scale assessing moral distress custom-tailored for OP LASER LTCF deployment.

*Resident/patient care compromised by:*

1. pressure to give aggressive/unnecessary treatment or tests.
2. pressure to not give necessary tests/treatments.
3. residents’ reported concerns going ignored.
4. resident care by unqualified staff
5. staff causing unnecessary suffering or not relieving pain/symptoms.
6. lack of health care provider continuity.
7. lack of support to report a violation of standards of practice or codes of ethics.
8. poor team communication.
9. insufficient information to ensure informed resident/patient consent.
10. too heavy of a resident/patient load.
11. lack of resources/equipment/bed capacity.
12. lack of administrative action/support.
13. fear of reprisal/reprimand if issues raised.
14. incomplete, unclear, or inconsistent treatment plans.
15. power hierarchies within teams, units, and institution.
16. inconsistent messages given to a patient.
17. lack of treatment with dignity and respect.
18. lack of staff adherence to hygiene rules and/or PPE use.

*Other potentially morally distressing situations you may have experienced…*

1. being isolated (i.e., quarantine) beyond what I believe is reasonable and without appropriate support from the CAF.
2. hostility from civilian staff directed at me and/or other CAF personnel.
3. civilian staff not demonstrating respect for deceased
4. civilian staff intentionally avoiding job responsibilities.
5. witnessing things that I knew were wrong but being unable to intervene due to CAF directives.
