# Supplementary material for: Moral Distress, Mental Health, and Risk and Resilience Factors Among Military Personnel Deployed to Long-Term Care Facilities During the COVID-19 Pandemic: Research Protocol and Participation Metrics
Source: JMIR Res Protoc. 2023 Nov 6;12:e44299. doi: 10.2196/44299 (PMC10629501; doi:10.2196/44299)
Supplement: Multimedia Appendix 3 [file resprot_v12i1e44299_app3.docx]

Table S3: Participant Demographic Breakdown for the Interview Study.

| **Variable** | **n** | **%** | **Expected % based on**  **T1 survey data** |
| --- | --- | --- | --- |
| **Op LASER LTCF Role** |  |  |  |
| Clinical health services personnel inside LTCF | 20 | 37.7% | 36% |
| Non-clinical health services personnel inside LTCF | 9 | 17.0% | 19% |
| Non-clinical health services personnel outside LTCF | 3 | 5.7% | 7% |
| Support Roles involved in Op LASER (e.g., HQ/Command, etc.) | 16 | 30.2% | 32% |
| Other | 2 | 3.8% | 5% |
| Blank | 3 | 5.6% | 0% |
| **Rank** |  |  |  |
| Junior NCM | 32 | 60.4% | 67% |
| Senior NCM | 7 | 13.2% | 13% |
| Junior Officer | 8 | 15.1% | 17% |
| Senior Officer | 6 | 11.3% | 4% |
| **Gender** |  |  |  |
| Women | 14 | 26.4% | 26% |
| Men | 39 | 73.6% | 73% |
| **Component** |  |  |  |
| Regular Force | 24 | 45.3% | 48% |
| Reserve Force | 28 | 52.8% | 52% |
| Unknown | 1 | 1.9% | 0% |
| **Province of Deployment** |  |  |  |
| Ontario | 18 | 34.0% | 34% |
| Quebec | 35 | 66.0% | 66% |
| **Age** |  |  |  |
| 17-29 | 24 | 45.3% | 56.1% |
| 30-39 | 15 | 28.3% | 29.6% |
| 40-49 | 10 | 18.9% | 10.7% |
| 50+ | 4 | 7.5% | 3.5% |
| **Family Status** |  |  |  |
| Single (never married) | 27 | 50.9% | 56% |
| Married/Common Law | 22 | 41.5% | 40% |
| Separated/Divorced/Widowed | 4 | 7.6% | 4% |
