# Supplementary material for: Moral Distress, Mental Health, and Risk and Resilience Factors Among Military Personnel Deployed to Long-Term Care Facilities During the COVID-19 Pandemic: Research Protocol and Participation Metrics
Source: JMIR Res Protoc. 2023 Nov 6;12:e44299. doi: 10.2196/44299 (PMC10629501; doi:10.2196/44299)
Supplement: Multimedia Appendix 4 [file resprot_v12i1e44299_app4.docx]

Multimedia Appendix 4

Table S4. Interview discussion domains.

| **DEMOGRAPHICS** |
| --- |
| - Age |
| - Sex/ Gender |
| - Relationship/ Household Status |
| - Elderly parents/relatives; at home or in a long term care facility |
| **MILITARY BACKGROUND** |
| - Regular Forces or Reserve Force |
| - Rank category |
| - MOC/Trade |
| - Years of military service (grouped) |
| - Role in Op LASER |
| **PRE-DEPLOYMENT EXPERIENCES** |
| - Think about the 2-3 weeks leading up to Op LASER. What was life like for you at work (or telework), and at home? |
| - What did you expect as you were getting deployed? |
| - How would you describe the CAF training and preparation for your role in this deployment (including your training but not limited to it)? Was it adequate? Overall CAF preparation and training for Op LASER? |
| **DEPLOYMENT EXPERIENCES** |
| - What were your leisure activities during your deployment at Op LASER, did you have the time to do any? (e.g., reading, movies, sports, entertainment, music, distance learning, etc.); were these activities helpful, did they help you to decompress on a daily or weekly basis? - Did you feel that any regulations were too restrictive or negatively affected your ability to decompress? - How quick was CAF able to facilitate leisure and recovery activities in the peak of the pandemic, and while deployed? |
| - a) What did you experience during your deployment? How did you feel about your experiences during Op LASER, positive and/or negative? - b) When did you experience this (name the event/emotion)? Did it happen before, during, after the deployment? How do you feel now about this emotion, now that it is over? |
| - If you place yourself back in time when you were serving in Op LASER, was there something, an event, a memory that really stands out, positive or negative? |
| - How was your relationship with other service members deployed with you during the Operation? |
| - Did you have a sense of a chain of command, of a command structure? - Did you follow something similar to “rules of engagement” for Op LASER? Did you have confidence that they would protect you against COVID-19 and help you do your job? |
| **POST DEPLOYMENT EXPERIENCES** |
| - What was you experience like after Op Laser ended? [potential prompts : did they go on another tasking or back to garrison, or back home; perceptions of support, e.g., immediate family, civilian and military community, CoC etc.; barriers to care] |
| - a) Is there something you wish were different about your experience? - b) Are there some things that were really positive about Op LASER that you are proud of and make you feel good? |
| - Did you experience anything that challenged your view of who you are, the world you live in; could you talk a bit more about this experience? - Did anything occur that might have affected or change your sense of right and wrong? |
| - Did the mission affect you in any positive ways (prompts: such as your appreciation for the value of life, your ability to empathize with others? Gratitude for relationships with family members? - Impact on spirituality or religious beliefs? |
| - Did your vision of your military service within CAF change after this deployment, how so, why? |
| - How did Canadians react to your service in Op Laser? - How were you perceived, yourself, by the people you were helping? - c) How were you treated by your family, friends, neighbours, community? |
| - How was your return to ‘normal, everyday life’ at home? |
| - Did Op LASER change how the military recognized your contribution? |
| - Did the LTCF reports that circulated in the media (tv, internet, news) influence your perception of what you were doing, of the operation? If so how? |
| - Is there anything, in that deployment that you wish was different; In terms of lessons learned, what do you want to make sure that the chain of command knows for next time. |
| - Did you want to add anything else, is there a question we forgot to ask? |
