# Supplementary material for: Moral Distress, Mental Health, and Risk and Resilience Factors Among Military Personnel Deployed to Long-Term Care Facilities During the COVID-19 Pandemic: Research Protocol and Participation Metrics
Source: JMIR Res Protoc. 2023 Nov 6;12:e44299. doi: 10.2196/44299 (PMC10629501; doi:10.2196/44299)
Supplement: Multimedia Appendix 5 [file resprot_v12i1e44299_app5.docx]

Multimedia Appendix 5

Table S5. Distribution of Survey Completion Time for T1, T2, and T3.

| Quantiles | Completion Time (min) | | |
| --- | --- | --- | --- |
|  | T1 | T2 | T3 |
| 0% | 5.88 | 3.60 | 6.75 |
| 5% | 17.11 | 9.66 | 11.12 |
| 10% | 20.23 | 12.08 | 14.46 |
| 15% | 22.90 | 13.72 | 15.55 |
| 20% | 24.77 | 15.24 | 16.98 |
| 25% | 26.80 | 17.40 | 18.68 |
| 30% | 29.26 | 18.23 | 19.67 |
| 35% | 31.66 | 19.00 | 21.20 |
| 40% | 33.70 | 20.88 | 22.92 |
| 45% | 35.95 | 22.10 | 25.38 |
| 50% | 39.08 | 23.97 | 27.16 |
| 55% | 43.15 | 25.90 | 29.12 |
| 60% | 46.47 | 28.32 | 31.16 |
| 65% | 51.90 | 31.56 | 34.00 |
| 70% | 61.96 | 34.18 | 38.44 |
| 75% | 73.77 | 37.95 | 45.47 |
| 80% | 91.69 | 45.27 | 57.65 |
| 85% | 180.88 | 75.31 | 85.77 |
| 90% | 4875.90^1^ | 359.61^4^ | 227.32^7^ |
| 95% | 26258.10^2^ | 32648.30^5^ | 7936.77^8^ |
| 100% | 49420.70^3^ | 44780.87^6^ | 43374.98^9^ |

Equivalents:

^1^4875 min = 3.39 days

^2^26258 min = 18.23 days

^3^49420 min = 34.32 days

^4^359 min = 5.99 hours

^5^32648 min = 22.67 days

^6^44780 min = 31.10 days

^7^227 min = 3.78 hours

^8^7937 min = 5.51 days

^9^43375 min = 30.12 days
